# Paraneoplastic Antigen Ma2 Autoantibodies as Specific Blood Biomarkers for Detection of Early Recurrence of Small Intestine Neuroendocrine Tumors

**DOI:** 10.1371/journal.pone.0016010

**Published:** 2010-12-30

**Authors:** Tao Cui, Monica Hurtig, Graciela Elgue, Su-Chen Li, Giulia Veronesi, Ahmed Essaghir, Jean-Baptiste Demoulin, Giuseppe Pelosi, Mohammad Alimohammadi, Kjell Öberg, Valeria Giandomenico

**Affiliations:** 1 Department of Medical Sciences, Endocrine Oncology, Uppsala University, Uppsala, Sweden; 2 Department of Medical Sciences, Uppsala University Hospital, Uppsala, Sweden; 3 Division of Clinical Immunology, Uppsala University, Uppsala, Sweden; 4 Division of Thoracic Surgery, European Institute of Oncology, Milan, Italy; 5 de Duve Institute, Université Catholique de Louvain, Brussels, Belgium; 6 Division of Pathology and Laboratory Medicine, European Institute of Oncology and University of Milan School of Medicine, Milan, Italy; 7 Department of Medical Sciences, Science for Life Laboratory, Uppsala University Hospital, Uppsala, Sweden; University of Muenster, Germany

## Abstract

**Background:**

Small intestine neuroendocrine tumors (SI-NETs) belong to a rare group of cancers. Most patients have developed metastatic disease at the time of diagnosis, for which there is currently no cure. The delay in diagnosis is a major issue in the clinical management of the patients and new markers are urgently needed. We have previously identified paraneoplastic antigen Ma2 (PNMA2) as a novel SI-NET tissue biomarker. Therefore, we evaluated whether Ma2 autoantibodies detection in the blood stream is useful for the clinical diagnosis and recurrence of SI-NETs.

**Methodology/Principal Findings:**

A novel indirect ELISA was set up to detect Ma2 autoantibodies in blood samples of patients with SI-NET at different stages of disease. The analysis was extended to include typical and atypical lung carcinoids (TLC and ALC), to evaluate whether Ma2 autoantibodies in the blood stream become a general biomarker for NETs. In total, 124 blood samples of SI-NET patients at different stages of disease were included in the study. The novel Ma2 autoantibody ELISA showed high sensitivity, specificity and accuracy with ROC curve analysis underlying an area between 0.734 and 0.816. Ma2 autoantibodies in the blood from SI-NET patients were verified by western blot and sequential immunoprecipitation. Serum antibodies of patients stain Ma2 in the tumor tissue and neurons. We observed that SI-NET patients expressing Ma2 autoantibody levels below the cutoff had a longer progression and recurrence-free survival compared to those with higher titer. We also detected higher levels of Ma2 autoantibodies in blood samples from TLC and ALC patients than from healthy controls, as previously shown in small cell lung carcinoma samples.

**Conclusion:**

Here we show that high Ma2 autoantibody titer in the blood of SI-NET patients is a sensitive and specific biomarker, superior to chromogranin A (CgA) for the risk of recurrence after radical operation of these tumors.

## Introduction

Gastrointestinal neuroendocrine tumors (GI-NETs) by tradition are known as carcinoids and they are rare tumors. They arise from enterochromaffin cells, which are sparse neuroendocrine cells disseminated throughout the GI tract [1], [2], [3]. GI-NETs comprise well-differentiated NET (benign carcinoid), well-differentiated neuroendocrine carcinoma (malignant carcinoids) and poorly differentiated neuroendocrine carcinoma [4]. GI-NETs include small intestine neuroendocrine tumors (SI-NETs), which have been called midgut carcinoids [5]. NETs are life-threatening diseases that have been the subject of investigation for more than a century. They derive from cells that have the unique ability to synthesize, store and secrete a variety of metabolic active products peptides, and amines, which cause specific clinical syndromes in different parts of the body [3], [6]. Lung NETs comprise 20% of all lung cancers and represent a spectrum of tumors differentiating from neuroendocrine cells of the respiratory tract. They are managerially separated into four subgroups on the basis of clinical characteristics: typical carcinoid tumor (TC), atypical carcinoid tumor (AC), large-cell neuroendocrine carcinoma (LCNEC), and small-cell lung carcinoma (SCLC) [7], [8].

Most GI and lung NET patients have developed metastatic disease at the time of diagnosis and surgery is seldom curative [2], [9]. Surgical debulking and hepatic embolization are not curative *per se* and conventional chemo-radiotherapy has little or no effects. The current treatments of metastasized GI and lung NETs aim at controlling tumor growth and hormonal secretion by using mainly somatostatin analogs and interferon alfa [10]. The slow progress in the development of novel curative treatments is partly due to a lack of tumor biology knowledge, late diagnosis, and a lack of novel biomarkers for early tumor detection and recurrence [11]. Today the best-characterized circulating biomarker that identifies NETs in general is chromogranin A (CgA) which belongs to the granin family. The family counts eight members [6]. Furthermore, CgA had been considered the first important circulating biomarker to evaluate recurrence in radically operated midgut carcinoid tumors [12] which, herein are classified as SI-NETs.

We have recently identified six novel marker genes for neuroendocrine tumor cells by using Affymetrix microarrays analysis [13]. We profiled normal small intestine mucosa, primary tumors and liver metastasis using advanced bioinformatics analysis to identify differentially and specifically expressed genes. One of the novel marker genes found was *PNMA2*. These results have been deposited on NCBI's GEO (accession number: GSE9576) and EBI's Array-Express database (accession number: E-TABM-389) [13]. Human *PNMA2* encodes the paraneoplastic antigen Ma2 which belongs to the human PNMA family [14]. Paraneoplastic antigens, which are normally expressed only in neuronal tissues, can in the process of carcinogenesis be detected in tumors located outside the nervous system. The term paraneoplastic syndrome (PNS) refers to a pathology caused by tumor cells, which systematically produce a large amount of hormones, growth factors, cytokines and a variety of specific symptoms [15], [16].

PNS may affect any part of the nervous system and muscles. Immunoresponses to cancer, which cross-react with self-antigens in the nervous system or muscle lead to production of onconeuronal antibodies detection [16], [17]. Despite the efforts to elucidate the effects of such antibodies on neurons, only a few onconeuronal antibodies have been identified as primary effectors of neurological symptoms. PNS is uncommon and defined by an acute or sub-acute neurological syndrome associated with a cancer. Examples of PNS are sub-acute cerebellar ataxia, limbic encephalomyelitis, Lambert-Eaton myasthenic syndrome, dermatopolymyositis and intestinal pseudo- obstruction [15]. The symptoms of PNS often appear before the diagnosis of malignant cancer and probable cases of PNS may suggest early antitumor therapy and immunotherapy to prevent progressive neuronal death. Symptoms can be also caused by neuroendocrine cells that are present in the GI tract and in the lung [2]. However, understanding whether the antibodies are associated with specific neurological symptoms or are only marker of anticancer immune reaction is difficult [16].

The functions of the proteins encoded by PNMA genes are not clear. However, MOAP1/PNMA4 was identified as a Bax-associating protein inducing apoptosis in mammalian cells. The significant homology of MOAP1/PNMA4 and the other PNMA proteins suggested a potential role in apoptosis for PNMA proteins [18], [19]. Antitumor immune responses to neuronal antigens expressed by tumor cells may lead to detectable levels of antibodies in serum and plasma [14], [15], [16].

Voltz et al. identified anti-Ma2 antibodies in patients suffering from testicular cancer and paraneoplastic limbic or brain-stem encephalitis or both [20]. The antibodies are often present in sera from patients suffering from neurological PNS [15], [21], [22]. Scientific evidence has shown that anti-Ma2 positive sera sometimes are associated with tumor diagnosis [15], [16], [23], [24].

The finding that Ma2 is expressed in primary SI-NETs and metastases [13] prompted us to screen whether Ma2 autoantibodies are detectable in blood of NET patients to establish potential novel biomarkers and to evaluate their clinical implications in tumor diagnosis and prognosis. In this study, we set up a novel indirect enzyme-linked immunosorbent assay (ELISA) to detect Ma2 autoantibodies in blood samples of 124 SI-NET patients at different stages of disease. We also extended the evaluation to lung carcinoid patients' blood to verify that our assay is able to detect the presence of Ma2 autoantibodies in different neuroendocrine tumors. SI-NETs and lung carcinoids originate from cells, which synthesize and secrete biologically active compounds that are able to produce either humoral PNS or less commonly neurological PNS. The clinicians at the Uppsala University Hospital and the European Institute of Oncology have never identified PNS or neurological symptoms in the patients we used in our study.

To our knowledge this is the first study that shows that Ma2 autoantibodies is a biomarker with diagnostic and prognostic relevance in the blood stream of SI-NET patients. Ma2 autoantibodies evaluation can identify recurrence of SI-NET patients with higher precision than measurements of plasma CgA levels. This novel finding can significantly improve the clinical management of SI-NET patients.

## Results

### Indirect ELISA detects Ma2 autoantibodies in primary SI-NET patients

**Figure 1:**
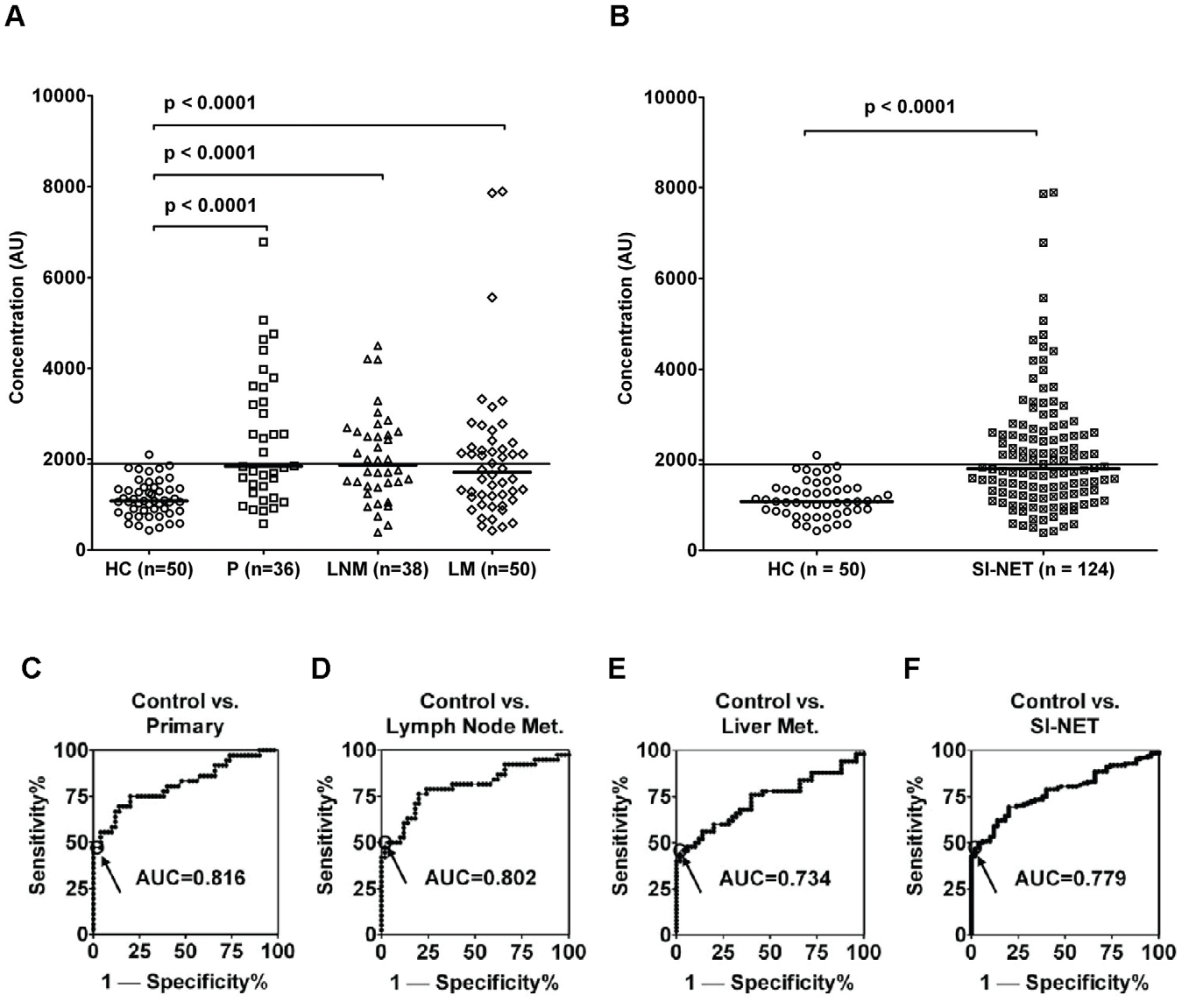
A novel indirect ELISA detects Ma2 autoantibodies in serum from SI-NET patients. Healthy controls (HC), untreated SI-NET patients with primary tumor (P), lymph node metastasis (LNM) and liver metastasis (LM) were evaluated for the presence of Ma2 autoantibodies. Horizontal bars in bold indicate the medians in each group. Horizontal lines indicate the cutoff at 1.96 SD above the mean of anti-Ma2 concentration of HC. Significant differences, which are shown by p values, were detected between HC and each stage of malignancy (A) and between HC and all the SI-NET patients (B). ROC analyses and AUCs results are shown in C, D, E and F. The highlighted points in each graph indicate the cutoff.

Significantly higher Ma2 autoantibody titer in SI-NET patients compared to healthy volunteers were detected by using the novel indirect ELISA. The reproducibility of the assay is expressed in intra- and inter-assay percent coefficient of variation (CV) as explained in Material & Methods. The difference was clear both considering the diverse categories of malignancies, primary tumors, lymph node and liver metastasis (Figure 1A), and the whole SI-NETs cohort of patients (Figure 1B). The sensitivity of the ELISA is from 46% to 50%. The specificity threshold is 98% as described in Material and Methods. Receiving operating characteristic (ROC) curve analyses were used to evaluate the possible use of Ma2 autoantibodies as early blood marker for SI-NET. ROC analyses show areas under the curves (AUCs) from 0.734 to 0.816 indicating good accuracy as a diagnostic test. Figure 1C, 1D and 1E show the results of healthy controls (HC) versus (vs.) SI-NET primary tumors (P), vs. SI-NET lymph node metastases (LNM) and vs. SI-NET liver metastases (LM). Figure 1F shows the results of HC vs. SI-NET patients as a whole, for all stages of disease. The results are summarized in the upper part of Table 1.

**Table 1:** Results of indirect ELISA assay for Ma2 autoantibodies.

Group	Type	Anti-Ma2 Median (Range) (AU)	Sensitivity (n)	Specificity (n)	AUC (95% CI, p*-* value)
Healthy Controls		1079 (433–2100)		98.0% (49/50)	
SI-NETs	Primary Tumor	1844 (579–6782)	47.2% (17/36)	−	0.816 (0.720–0.912, p<0.0001)
	Lymph Node Metastasis	1868 (391–4499)	50.0% (19/38)	−	0.802 (0.702–0.903, p<0.0001)
	Liver Metastasis	1712 (424–7890)	46.0% (23/50)	−	0.734 (0.633–0.835, p<0.0001)
	Total SI-NET	1804 (391–7890)	47.6% (59/124)	−	0.779 (0.712–0.846, p<0.0001)
Lung Carcinoids	TLC	1456 (570–6891)	30.8% (16/52)	−	0.693 (0.591–0.795, p = 0.0008)
	ALC	2013 (515–3506)	50.0% (7/14)	−	0.766 (0.592–0.939, p = 0.0025)
	Total Lung Carcinoids	1494 (515–6891)	34.8% (23/66)	−	0.709 (0.616–0.801, p = 0.0001)

### Detection of Ma2 autoantibodies in sera from HC and primary SI-NET patients

**Figure 2:**
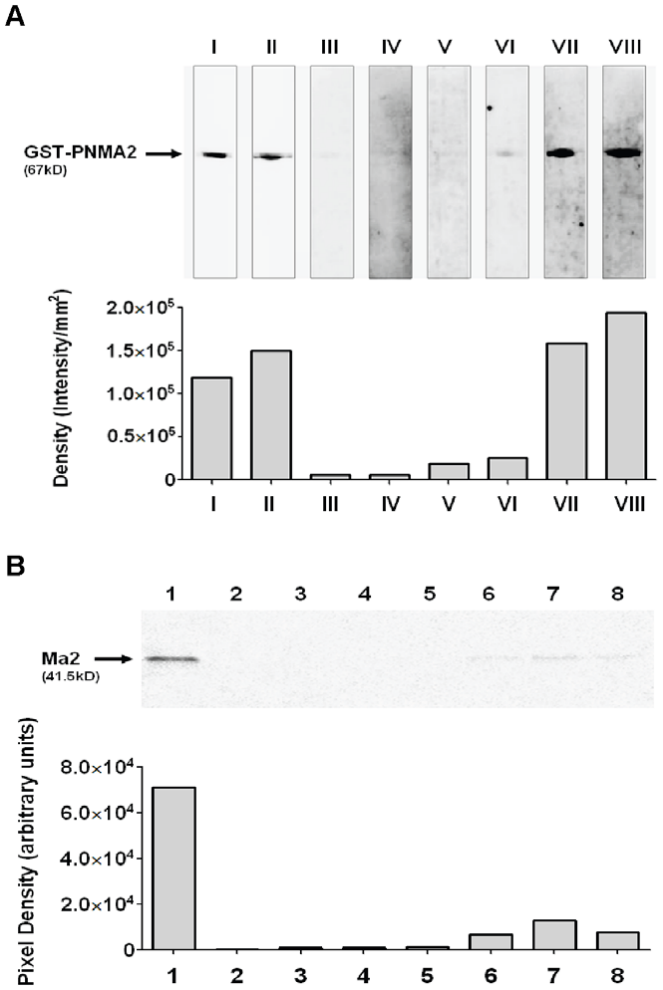
Detection of Ma2 autoantibodies in serum of healthy controls and primary SI-NET patients. 2A) GST-tagged fusion PNMA2 recombinant protein was subjected to SDS-PAGE followed by Western blotting analysis. A mouse anti-GST antibody (lane I) and a goat anti-PNMA2 antibody (lane II) verified the GST-tagged PNMA2 recombinant protein. Lanes III-IV, sera from 2 healthy donors; Lanes V-VI sera from 2 primary SI-NET patients with low anti-Ma2 titer; Lanes VII-VIII, sera from 2 primary SI-NET patients with high anti-Ma2 titer. Lanes III to VIII show differences in the amount of Ma2 autoantibodies. The lower panel shows the semiquantitative measurement of band densities of the blot. 2B) autoradiographic image of sequentially immunoprecipitated ^35^S-Met-Ma2, positive control (lane 1); negative control (lane 2); sera from 3 healthy donors with low anti-Ma2 titer (lanes 3–5); sera from 3 primary SI-NET patients with high anti-Ma2 titer (lanes 6–8). The lower panel shows the semiquantitative measurement of pixel densities of the autoradiographic image.

The ELISA results inspired us to further characterize the presence and specificity of autoantibodies to Ma2 in serum of SI-NET patients with primary tumor. The presence was verified by using western blot analysis and the specificity by using sequential immunoprecipitation. We loaded purified GST-tagged PNMA2 recombinant protein on SDS-PAGE and blotted the gel. The Western blot membrane was immunoblotted either with commercial antibodies or serum samples. We confirmed that our commercial goat antibody specifically detects Ma2 antigen (Figure S1). To confirm the purity of GST-tagged PNMA2 recombinant protein anti-GST- and anti-PNMA2 antibodies were used as controls (Figure 2A, lanes I and II). Sera from healthy controls exhibit minimum titer of Ma2 autoantibodies, determined by the indirect ELISA and shown by the low recognition of GST-tagged PNMA2 recombinant protein (lanes III and IV). Primary SI-NET patients, with low titer of Ma2 autoantibodies, determined by the indirect ELISA, showed low recognition of GST-tagged PNMA2 recombinant protein (lanes V and VI), whereas SI-NET patients with high frequency of Ma2 autoantibodies showed high recognition (lanes VII and VIII). The lower panel of Figure 2A shows the semiquantitative measurement of band densities.

Next we confirmed the specificity of Ma2-specific autoantibodies in serum samples from the patients by sequential immunoprecipitation using ^35^S-methionine-labeled human Ma2 protein generated by *in vitro* transcription-coupled translation as described in Material and Methods. Ma2-specific autoantibodies were detected in serum samples from primary SI-NET patients expressing high titer of Ma2 autoantibodies (Figure 2B, lanes 6, 7, and 8) but not in serum samples from healthy controls (lanes 3, 4, and 5). Lane 1 shows immune precipitation with a commercial goat anti-human PNMA2 (positive control) and lane 2 shows immunoprecipitation without antibody or serum (negative control). The lower panel of Figure 2B shows the result of autoradiography of the blot. Recently, we showed that serum antibodies of patients detect Ma2 and faintly Ma1 by using commercial immuno-dot-blot (Figure S1).

### Progression free survival (PFS) and recurrence free survival (RFS) of primary SI-NET patients, after surgery with curative intent, depend on Ma2 autoantibody titer

**Figure 3:**
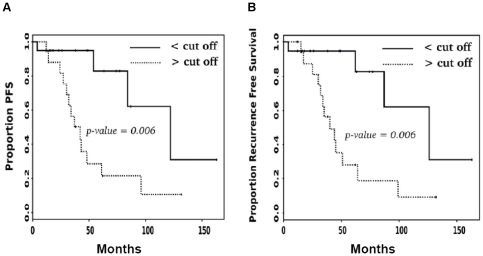
PFS and RFS of primary SI-NET patients after surgery with curative intent depend on Ma2 autoantibody titer. Patients were divided in two groups based on the Ma2 autoantibody titer either below or above the cutoff at 1900 AU as described in Material and Methods. Kaplan-Meier survival curve analyses were plotted for PFS (A) and RFS (B). The p-values of the differences between the two groups were obtained by using the log-rank test for each evaluation.

We have evaluated the clinical data of 36 patients followed up after radical operation of primary tumors with a curative intent. We evaluated Kaplan-Meyer survival curves, followed by a log-rank test to determine whether the curves were different. The hazard ratios were calculated, based on Cox regression function as described in Material and Methods, and found to be 4.31 (p-value = 0.011) for progression free survival (PFS) and 4.24 (p-value = 0.012) for recurrence free survival (RFS). The analysis showed that 19 patients with Ma2 autoantibody titer below the cutoff had a longer PFS compared to 17 patients with Ma2 autoantibody titer higher than the cutoff, Figure 3A. The same was true for patients with RFS, Figure 3B. The significance of the analyses is clearly expressed by both p-values = 0.006. The median survival time for each group of patients was estimated from the survival curves. It was clearly shorter for patients with Ma2 autoantibody titer higher than the cutoff with an estimated time of about 40 months compared to those with levels below the cutoff with an estimated survival time of about 125 months. The results are summarized in Table 2. Clinical information for patients with Ma2 autoantibodies concentration < cutoff are to the left in Table 2 and clinical information for patients with Ma2 autoantibodies concentration > cutoff are to the right.

**Table 2:** Ma2 autoantibody levels and associated clinical characteristics of primary SI-NET patients analyzed after surgery with a curative intent.

Anti-Ma2 Concentration < cutoff						Anti-Ma2 Concentration > cutoff					
Age/Gender	Anti-Ma2 (AU)	CgA (ng/ml)	Follow-up (month)	PFS (month)	Recurrence (month)	Age/Gender	Anti-Ma2 (AU)	CgA (ng/ml)	Follow-up (month)	PFS (month)	Recurrence (month)
47/M	915	3.7	63	63		43/M	6785	2.6	42	24	25
77/M	1091	2.6	163	163		37/F	4402	2.9	132	132	
67/F	1686	6.4	146	122	126	35/M	3200	2.4	62	62	
76/M	887	4.2	96	84	87	70/M	2548	2.8	144	96	99
64/M	1645	2.1	38	38		70/F	4758	3.4	96	48	51
59/M	1596	8.9	6	4	4	75/M	5064	3.0	102	61	64
52/F	1148	5.5	23	23		53/M	3982	3.3	80	12	15
50/F	1445	3.5	15	15		54/M	2547	2.6	19	14	17
68/M	1404	4.7	12	12		47/M	2553	3.8	91	42	44
38/M	1582	2.9	18	18		54/F	3794	3.5	44	34	35
70/M	1058	2.6	16	16		75/F	2456	9.2	12	12	
62/M	579	1.8	87	87		30/F	3005	13.6	52	37	40
71/M	1255	3.2	78	54	62	76/M	3260	4.3	38	38	
69/M	1814	2.2	77	77		67/M	2152	1.8	49	27	30
48/F	1736	3.3	49	49		67/M	3581	1.6	61	43	45
67/M	964	1.9	48	48		71/M	4640	4.5	75	30	32
78/M	1835	3.7	26	26		62/M	3612	2.1	43	32	34
82/M	862	3.7	18	18							
76/F	1852	2.5	74	74							
**Median**											
**67**	**1404**	**3.3**	**48**	**48**	**74.5**	**62**	**3581**	**3.0**	**62**	**37**	**35**
**Mean**											
**64.3**	**1334.4**	**3.7**	**55.4**	**52.2**	**69.8**	**58**	**3667**	**4.0**	**70.7**	**43.8**	**40.8**

Circulating CgA is considered important in indicating tumor recurrence in most radically operated midgut carcinoid tumor patients. Therefore, we wanted to evaluate the significance of CgA protein expression levels on PFS and RFS in our patient material. The median level of circulating CgA in 36 patients with primary tumors was mainly within the same reference range with CgA <4.0 ng/ml, as shown in Table 2. The Cox regression modeling as explained in Material and Methods revealed that anti-Ma2 titer is more predictive of patient outcome than CgA concentration. The statistical analysis of PFS showed that Cox proportional hazard for the progression time in function of anti-Ma2 + CgA properly fits; p value = 0.0192. Clear effect of anti-Ma2 titer was found (coefficient = 0.000409 and p-value = 0.013), whereas no significant effect of CgA was found (coefficient = 0.141492, p-value = 0.087). Similar analysis of RFS showed that Cox proportional hazard for the recurrence time in function of anti-Ma2 + CgA fits with p-value = 0.0222. Clear effect of anti-Ma2 titer was shown (coefficient = 0.000397, p-value = 0.015); whereas no significant effect of CgA was found (coefficient = 0.140190, p-value = 0.089). We observed that in the group of 19 patients with Ma2 autoantibody titer < cutoff only 4 patients had tumor recurrence during the follow-up. 2 of these 4 patients had increased CgA levels. Furthermore, in the group of 17 patients with Ma2 autoantibody titer > cutoff 13 patients presented tumor recurrence. CgA levels increased in only one out of these 13 patients. Our study clearly shows by Cox modeling that CgA is not predictive of PFS and RFS.

### Circulating Ma2 autoantibody levels and Ma2 expression in SI-NET patients

Matched blood samples and formalin-fixed paraffin-embedded tumor material from 20 untreated SI-NET patients at different stage of disease with primary tumors (P), lymph node metastasis (LNM) and liver metastasis (LM) were analyzed to detect a possible correlation between the serum titer of Ma2 autoantibodies and the tumor expression of Ma2 antigen. Patients are described in Table S1. We evaluated serum samples by ELISA and the expression of Ma2 by immunohistochemistry (IHC) analysis. The ELISA results found that 12 patients out of 20 expressed Ma2 autoantibodies with titer higher than the cutoff, whereas 8 patients out of 20 expressed Ma2 autoantibodies with titer lower than the cutoff. The results are summarized in Table S1. We confirmed that our commercial rabbit antibody specifically detects Ma2 antigen (Figure S1) Ma2 immunohistochemistry was then performed on primary tumors and metastases from the same patients. The results showed that all 12 patients who expressed Ma2 autoantibodies with titer higher than the cutoff were positive for Ma2 in the tumors. Moreover, 7 out of 8 patients who expressed Ma2 autoantibodies with titer lower than the cutoff were positively stained as well. Therefore, we conclude that there is no evident correlation between the Ma2 autoantibody titer and the amount of Ma2 expression. Immunohistochemical Ma2 staining of the tumor specimens are summarized in Table S1. Positively stained tissues showed PNMA2 immunoreactivity from faint to moderate granular accumulation of immunostaining product confined to the cytoplasm of most tumor cells. Stromal cells were completely negative verifying Ma2 immunoreaction specificity. Figure S2A shows four representative stainings from patients with high titer of Ma2 autoantibodies and Figure S2B shows four representative stainings from patients with low titer of Ma2 autoantibodies.

Patient serum is able to recognize Ma-2 and Ma-1 as shown in Figure S1. Immunohistochemistry analysis was also performed on paraffin sections from patients suffering from SI-NET primary tumors and liver metastases by using serum from patients with high titer of Ma2 autoantibodies and serum from healthy controls with low titer of Ma2 autoantibodies. The Auerbach's plexus, which is part of the enteric nervous system, is located between the longitudinal and circular layers of *muscularis externa* in the gastrointestinal tract and provides motor innervation. Serum from healthy controls faintly stained the tumor cells and the neurons whereas serum patients with high titer of Ma2 autoantibodies clearly detected Ma2 in the neuroendocrine tumor cells and neurons of the Auerbach's plexus (or myenteric plexus). Representative immunohistochemical Ma2 staining of tumor cells and neurons is shown in Figure S3.

### Novel indirect ELISA detects Ma2 autoantibodies in lung carcinoids

**Figure 4:**
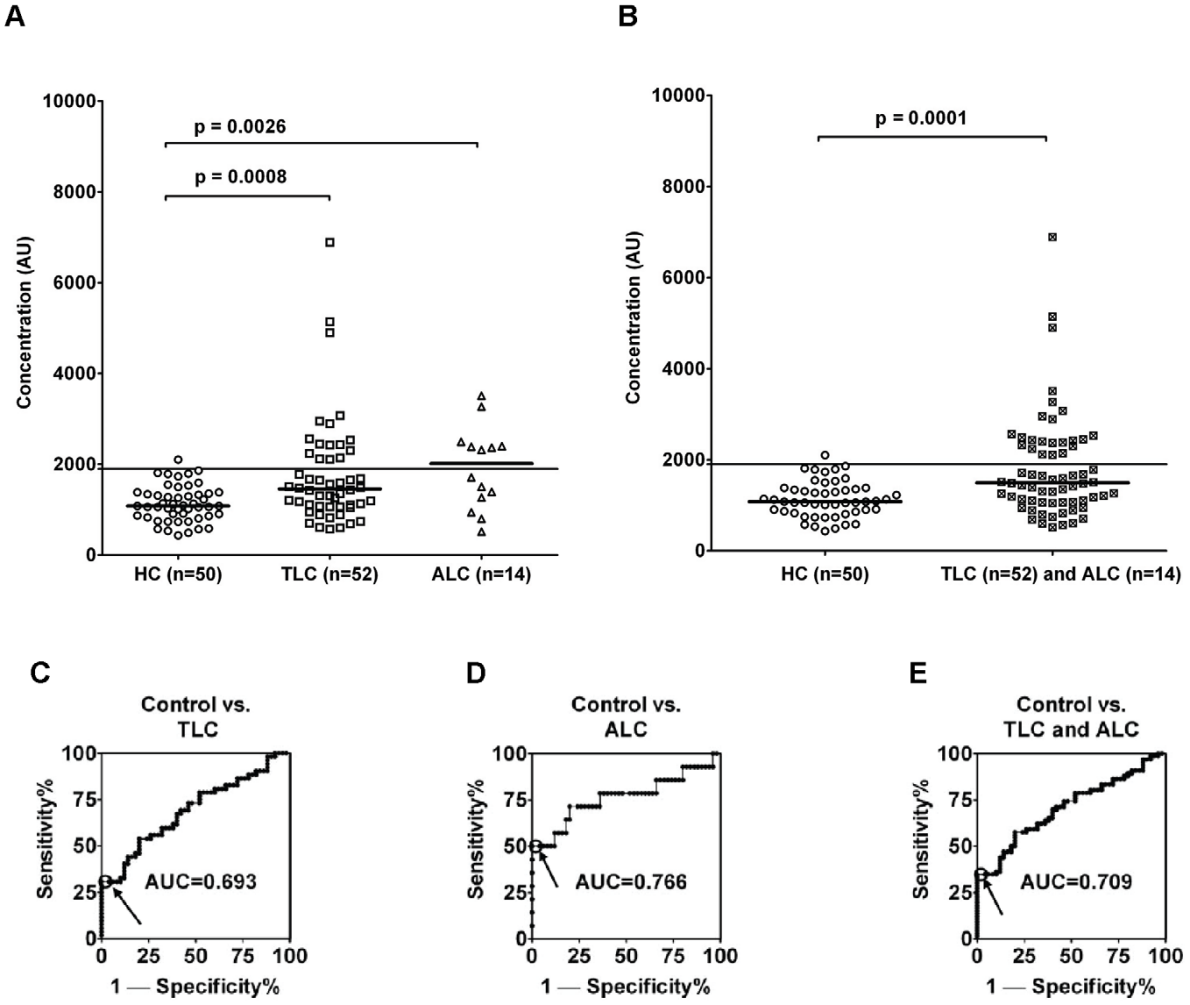
A novel indirect ELISA detects Ma2 autoantibodies in lung carcinoid patients. Healthy controls (HC, n = 50), untreated TLC (n = 52) and ALC patients (n = 14) were evaluated for the presence of Ma2 autoantibodies. Horizontal bars in bold indicate the medians in each group. Horizontal lines indicate the cutoff at 1900 AU (1.96 SD above control mean). 4A) p-values between healthy controls and the two groups of patients, TLC and ALC show significant differences. 4B) p-values between healthy controls and all lung carcinoids (TLC + ALC) show significant differences. ROC curve analyses and AUCs are shown in Figure 4C, 4D and 4E. The highlighted points in each graph indicate the cutoff.

Previous results on small cell lung carcinoma samples suggested analyzing the presence of Ma2 antigen in typical and atypical lung carcinoid tissue. Immunohistochemistry analysis on paraffin sections showed increased expression of Ma2 in comparison with normal internal tissues. The mean of Ma2-positive tumor cells in typical carcinoids is 54% and in atypical carcinoids is 28%, independent of tumor growth patterns of the former, Figure S4. We next explored whether lung carcinoid tumors were associated with anti-Ma2 antibodies. The 66 blood samples from lung carcinoids patients (52 typical and 14 atypical) were compared to 50 samples from healthy volunteers. The samples were first analyzed as 2 groups according to the TC and AC classification and named TLC and ALC, Figure 4A. The two different groups are significantly distinguished from healthy controls as shown by the p-values. Lung carcinoids were also considered as a whole and the results show that the group of patients is clearly distinguished from healthy controls as indicated by p-value in Figure 4B. The sensitivity, specificity and AUCs of ROC analysis in lung carcinoid patients are presented in the lower part of Table 1. ROC curve analysis evaluated the possible use of Ma2 autoantibodies as potential early blood marker for lung carcinoids. ROC analyses were used to study healthy controls vs. TLC as shown in Figure 4C, healthy controls vs. ALC as shown in Figure 4D, and healthy controls vs. both typical and atypical lung carcinoids, as shown in Figure 4E. The AUCs are between 0.693 and 0.766, indicating fair accuracy as a diagnostic test.

## Discussion

We have recently profiled primary SI-NETs and liver metastases by using Affymetrix microarrays and identified that paraneoplastic antigen Ma2 is produced by enterochromaffin cells and neuroendocrine tumor cells [13]. Few studies have correlated paraneoplastic syndrome to patients with midgut carcinoids and lung carcinoids as well [15], [25]. Our finding that Ma2 was consistently detected in primary SI-NET specimens and in a variety of metastasis made it interesting to investigate whether antibodies against Ma2 are present in the blood of SI-NET patients.

The main purpose of the present study was to assess whether Ma2 autoantibodies can be used as a specific and sensitive blood-based marker for a more accurate diagnosis and progression of SI-NETs. We set up a novel indirect ELISA able to accurately screen the Ma2 autoantibody levels in serum or plasma samples. We confirmed the specificity of serum Ma2 autoantibodies by using western blot and sequential immunoprecipitation analyses. A central aspect of our investigation is that Ma2 autoantibodies discriminate a large portion of SI-NET patients from healthy controls both considering the diverse tumor categories which reflect different stages of disease and as a whole. The evaluation of the different levels of Ma2 autoantibodies, which were detectable in the diverse categories of patients, is reliable considering the high values of sensitivity, specificity and AUC values from the ROC analyses. The AUC values are convincing to continue to screen more samples as new SI-NET patients are being diagnosed. This would imply that Ma2 autoantibodies are likely produced early in the development of SI-NETs, with maintenance of steady blood levels during tumor progression.

One of the most important clinical problems is to detect early disease as well as to detect early recurrence after surgery with a curative intent. Increased CgA levels in blood is the standard biomarker and considered important in indicating tumor recurrence in most radically operated midgut carcinoid tumor patients [12], [26]. We had the possibility to follow 36 patients with primary tumors operated with a curative intent. We found that the median level of CgA is within the reference range, <4 ng/ml in both groups of patients below and above cutoff level for Ma2 autoantibodies i.e. that CgA levels are not indicative in this group of patients. We observed that in the group of patients with Ma2 autoantibodies below cutoff level, 4 patients out of 19 recurred within a median of 74 months, whereas in the group of patients with Ma2 autoantibodies above cutoff level, 13 patients out of 17 recurred within a median of 35 months. Therefore, in this case the ELISA that detect Ma2 autoantibodies can reliably identify about 47% of the SI-NET patients at the primary stage of the disease and the levels of autoantibodies correlate with the progression and recurrence free survival of the patients.

Circulating levels of Ma2 autoantibodies did not seem to reflect the tumor mass *per se* as circulating levels for patients with lymph node or liver metastases with higher tumor load did not show significantly higher levels of autoantibodies. This supports the notion that Ma2 autoantibodies appear early during SI-NET development. These data pave the way for the possibility of using Ma2 autoantibodies as a reliable marker for tumor detection and progression of SI-NETs growth. Furthermore, the identification of Ma2 as a target of the autoimmune response in patients with SI-NET may provide the first insights into the molecular mechanisms of paraneoplastic syndrome in patients with these tumors. We also presented preliminary results dealing with TLC and ALC. All blood and tumor samples of lung carcinoids tested so far by ELISA and immunohistochemistry on paraffin sections showed increased expression of Ma2 in comparison with normal internal tissues. This supports the view that Ma2 protein accumulation and the presence of Ma2 autoantibodies is closely associated with the development of several types of differentiated NETs.

Our study shows that lower titer of Ma2 autoantibodies correlates to a lower probability of recurrence with longer survival of SI-NET patients. This finding contradicts those reported in other studies, for instance Graus et al. detected autoantibodies, denoted anti-Hu antibodies, which recognize antigens expressed by neurons, in small-cell lung carcinoma that have been associated to patients' longer survival [27]. Pujol et al. reported the presence of autoantibodies and spontaneous complete remission of a non-small cell lung cancer (SCLC) patient associated with anti-Hu syndrome. Moreover, like Graus et al, they concluded that the anti-Hu humoral immunology is associated to a positive tumor response [28]. However, a more recent study reported that onconeural antibodies, such as anti-Hu and anti-CV2/CRMP5 have a different behavior in different tumor types. Therefore, the prognosis of the same type of tumor may differ according to the type of analyzed onconeural antibodies [29].

The human immune system normally produces antibodies in response to foreign proteins, such those of pathogens, and ignores the body's own cells proteins to avoid to trigger disease. When the immune system fails to discriminate self from non-self, proteins start producing antibodies against self proteins denoted autoantibodies. A variety of theories have tried to explain why autoantibodies appear in different patient conditions. However, why humoral autoimmunity can cause diseases is not fully understood.

We detected Ma2 autoantibodies in the serum of SI-NET patients. However, PNS was not correlated to the analyzed SI-NET patients in this study. Although our conclusions differ from those from other groups, we speculate that the presence of high serum titer of Ma2 autoantibodies may predict poor prognosis in patients suffering from SI-NETs, as previously shown in different diseases. For instance, a significant study has evaluated autoantibodies against insulin and beta-islet cells in pancreatic adenocarcinoma. Conclusive data suggested that the high serum titer of both autoantibodies were associated with a worse outcome for the patients [30]. A second study evaluated several serum antibodies from colon cancer patients to isolate a novel cancer biomarker. The new isolated biomarker was aberrantly expressed and the level of serum autoantibodies was significantly higher in the patients than in matched non cancerous tissue in healthy donors. The authors of this study concluded that the detection of serum antibody to tumor antigens might be a better marker than serum antigens [31]. A third study has also showed that autoantibodies predict poor prognosis in patients with advanced esophageal squamous cell carcinoma [32].

Despite different reported findings we believe that the description of the relationship between the presence of Ma2 autoantibodies and progression/recurrence free survival in SI-NET offers new insights into the pathophysiology of this malignancy. Furthermore, we have also shown that the patient serum with high titer of Ma2 autoantibodies clearly detects Ma2 in the neuroendocrine tumor cells and neurons of the Auerbach's plexus (or myenteric plexus). The novel finding suggests exploring the potential role of anti-Ma2 in gut dysmotility via autoimmune-mediated neuronal apoptosis and the recent detection of anti-Ma1 antibodies is opening a new window to evaluate autoimmunity in the pathophysiology of these malignancies.

However, in conclusion we examined the performance characteristics of a novel ELISA to detect Ma2 autoantibodies in blood of SI-NET patients by measuring the sensitivity and specificity capacity in discriminating patients from healthy individuals. Furthermore, the presence of Ma2 autoantibodies is an earlier and more sensitive circulating marker than CgA for the risk of recurrence of the disease. To our knowledge this is the first novel NET biomarker after many years to be evaluated for diagnosis and risk of early recurrence after operation of primary SI-NET tumors with a curative intent.

## Materials and Methods

### Ethics Statement

All patient and control blood samples were included in the study after a written consent statement was obtained from each individual. The study was performed in accordance with the regional ethical committee at the Clinic of Endocrine Oncology, Uppsala University Hospital, Sweden (ref. no. 2005:241) and the internal revision board (IRB) of the European Institute of Oncology, Milan, Italy.

### Serum and plasma sample collection

Serum and plasma samples were obtained and analyzed from 124 patients with histopathology confirmed diagnosis of SI-NET: 36 with primary tumors, 38 with lymph node metastasis and 50 with liver metastasis. Patients with primary SI-NETs were collected after surgery with a curative intent. Serum and plasma samples were obtained and analyzed from 66 lung carcinoid patients: 52 TC (named TLC) and 14 AC (named ALC). The median ages of the patients were SI-NET 63 years (range, 27–82) and TLC & ALC 59 years (range, 13–81). Moreover, 50 serum samples from healthy volunteers were collected at the Uppsala University Hospital and used as negative controls. Clinicians at the Uppsala University Hospital, Neuroendocrine Center of Excellence, Uppsala, Sweden and at the European Institute of Oncology, Milan, Italy that follow the SI-NET and lung carcinoid patients have never identified PNS and neurological symptoms.

### Reagents and antibodies

Maxisorp strips (NunC, Roskilde, Denmark), GST-PNMA2 recombinant protein (Abnova, Taipei, Taiwan); 3,3′,5,5-tetramethylbenzidine (TMB) + substrate (Dako, Glostrup, Denmark), Peroxidase with dakocytomation peroxidase block (Dako), Dakocytomation envision® system labeled polymer-HRP anti-rabbit kit and 3-3′-diaminobenzidine (Dako), Mayer's hematoxylin (Histolab Product AB, Gothenburg, Sweden), Graded alcohol (Kemetyl, Vestby, Norway), Xylen (Solveco, Rosersberg, Sweden), Pertex® (Histolab, Gothenburg, Sweden), Tris-Glycine blotting buffer (Amresco, Solon, OH), Western blotting (WB) reagent and Lumi-Light WB substrate (Roche, Basel, Switzerland), EasyTag Methionine-L-^35^S, NEG709A005MC (PerkinElmer, Waltham, MA), Full-length cDNA clone for human PNMA2, ID6580976 (BioScience Geneservice, Cambridge, UK), TnT® SP6 Quick Coupled Transcription/Translation System (Promega, Madison, WI), Protein G-Sepharose beads (GE Healthcare, Little Chalfont, Buckinghamshire, UK), Peroxidase (HRP)-conjugated rabbit anti human IgG (anti-IgG) (Dako), rabbit polyclonal antibody anti-PNMA2 (Atlas Antibodies, Stockholm, Sweden), monoclonal mouse anti-GST, sc-138, polyclonal goat anti-human Ma2, sc-68099, HRP-donkey anti-goat, sc-2020, (Santa Cruz Biotechnology, Santa Cruz, CA), HRP-goat anti-rabbit, P0448, (Dako) and Ravo PNS-Blot, (Ravo Diagnostika GmbH, Freiburg, Germany).

### Indirect ELISA

We set up a novel indirect ELISA to detect Ma2 autoantibody levels in sera and plasma of NET patients and healthy volunteers. The standard curves to screen both serum and plasma samples were constructed by serial dilutions 1∶100, 1∶200, 1∶400, 1∶800, 1∶1,600, 1∶3,200 and 1∶6,400 using the serum and the plasma from the same patient with primary SI-NET, expressing anti-Ma2 used as reference. Two controls were used to evaluate individual runs. One was from a patient with primary SI-NET, expressing high titer of anti-Ma2 and the other one was from a healthy donor expressing low titer of anti-Ma2. GST-PNMA2 recombinant protein (Abnova, Taiwan), diluted in the coating buffer (15 mM Na2CO3, 35 mM NaHCO3, pH 9.6), at the concentration of 1 µg/ml was used to coat Maxisorp strips with 50 µl each well for 17 hours at 4°C. Strips were washed 3 times with washing buffer (PBS pH 7.4, 0.05% Tween-20). Strips were blocked with 200 µl of blocking buffer (PBS pH 7.4, 0.05% Tween-20 and 5% BSA) for 2 hours at room temperature. Strips were washed 3 times. Serum and plasma samples were diluted 1∶400 in PBS pH 7.4, 0.05% Tween-20, 1% BSA and loaded into wells. The samples were run in duplicates. They were incubated for 2 hours at room temperature. Strips were washed 6 times. Next, 50 µl of HRP-conjugated rabbit anti human IgG (1∶500) was added and incubated for 1 hour at room temperature. Strips were washed 8 times and then incubated with 100 µl TMB substrate for 30 min. Reaction was stopped with 100 µl of 1M H2SO4. Absorbance was read by Multiskan Ascent microplates photometer (Thermo Fisher Scientific, Waltham, MA) by using Ascent software version 2.6 (Thermo Elelectron corporation, Waltham, MA). Blank absorbance was subtracted and a 4-parameter logistic fitting standard curve was plotted. Serum and plasma reference samples were arbitrarily defined as 3200 arbitrary units (AU) for the standard curve and the results were expressed as concentrations according to the standard curve. The cut-off at 1900 AU (arbitrary unit) was chosen as 1.96 SD above the mean anti-Ma2 concentration of the healthy control group. Experiments were repeated two times. The precision of ELISA is expressed in intra- and inter-assay percent coefficient of variation (CV%). The intra-assay CV% between each duplicate is below 10%, a value that is considered to be reliable. The inter-assay CV% is 9.4% for the higher control and 8.9% for the lower control.

### Statistical Analysis

The power and sample size calculations were performed by using R.V. Lenth's Java applets for power and sample size [33]. The nonparametric Mann-Whitney *U* test [34] was used to assess the statistical significance of the anti-Ma2 concentration difference between the healthy controls and the different patient groups. The ROC curves provide a display of the relationships between the true positive rates (sensitivities) and false positive rates (1 − specificities) related to all binary tests for the biomarker. The shapes of the curves and AUCs presented with corresponding 95% confidence intervals (CI) are used to discriminate the diagnostic accuracies between tests [35]. The ROC curves were in this study generated to compare the AUCs and the predicted sensitivities and specificities among different NETs and different stages of disease. The statistical analyses were two-tailed and performed by using GraphPad Prism 5 (GraphPad Software, La Jolla, CA). We also computed Kaplan-Meier estimates for PFS and RFS [36] for two groups of patients based on the anti-Ma2 cutoff, as described above. Kaplan-Meier curves were compared by means of the log-rank test. The hazard ratio in survival analysis is the effect of an explanatory variable on the hazard or risk of an event, and such models include the Cox semi-parametric proportional hazards model. The hazard ratios for patients based on the diverse groups were calculated by Cox's regression method. Cox regression is a statistical technique that is used to determine the relationship between survival and several independent variables. This function provides better estimates of survival probabilities and cumulative hazard than those provided by the Kaplan-Meier function. We used a multivariate Cox proportional-hazards regression modeling to evaluate the effect of Ma2 autoantibody titer and CgA concentration on PFS and RFS of our patients [37]. Survival data were computed by using the survival package in R software. All p values <0.05 were considered significant.

### Immunohistochemistry

Formalin-fixed paraffin-embedded material with histopathologically confirmed diagnosis of metastasized SI-NETs and lung carcinoids were obtained as 3-µm-thick sections from the Pathology Biobank, Uppsala University Hospital and the European Institute of Oncology, Milan, Italy. We selected tumor material from 20 untreated SI-NET patients at different stage of disease with primary tumors (P), lymph node metastases (LNM) and liver metastases (LM). Tumor samples matched with blood samples used for ELISA. We selected both primary tumors and metastases from 14 lung carcinoid patients, 10 with TLC (1 man and 9 women) and 4 with ALC (3 men and 1 woman). Slides were deparaffinized in xylene, hydrated in graded alcohols and blocked for endogenous peroxidase with dakocytomation peroxidase block (Dako). For antigen retrieval slides were immersed in 10 mM citrate buffer, pH 6 and then boiled in microwave. Sections were stained with the primary rabbit polyclonal antibody anti-PNMA2 (Atlas Antibodies), diluted 1∶350 at 4°C overnight (O.N.). The day after, they were incubated with a secondary antibody by using HRP anti-rabbit kit (Dako), according to the manufacturers' instructions. Peroxidase activity was developed with 3-3′-diaminobenzidine (Dako). Sections were then counterstained in Mayer's hematoxylin (Histolab Product AB), dehydrated in graded alcohol and xylen and mounted by using Pertex (Histolab). The specificity of all immunoreactions was double-checked by substituting the primary antibody with a non-related polyclonal antibody at a comparable dilution, and with normal serum alone. Furthermore, serum from SI-NET patient and healthy control were used to stain Ma2 on five SI-NET tissue slides. The slides were blocked with peroxidase and then with 10% of normal rabbit serum for 30 min. Serum from one SI-NET patient with high titer of anti-Ma2 and from one healthy donor with low titer of anti-Ma2 were diluted 1∶100 in PBS and used to immunostain the slides at 4°C, O.N. After proper washing, HRP-conjugated rabbit anti-human IgG (1∶500, Dako) was applied for 30 min on the slides. Peroxidase activity was measured as described above.

### Western Blot Analysis

GST-tagged PNMA2 recombinant protein (Abnova) (440 ng in 2 µl) were mixed with an equal volume of 4X loading buffer (200 mM pH 6.8 Tris-HCl, 8% sodium dodecyl sulfate, 40% glycerol and 0.1% bromophenol blue) and 5% β-mercaptoethanol. The samples were heated at 95°C, loaded and run on a 10% sodium dodecyl sulfate-polyacrylamide gel electrophoresis (SDS-PAGE). The proteins were then blotted onto a nitrocellulose membrane in Tris-Glycine blotting buffer (Amresco) with methanol at 100 V for 2 hours at 4°C. After blotting the membrane was stained with Pouceau S to verify protein transfer. The membrane was cut in eight strips. The strips were blocked with TBS, 1% western blotting reagent (Roche) at 4°C, O.N. Each strip was then incubated at 4°C, O.N. with either a commercial antibody or a serum sample diluted in dilution buffer (TBS, 0.5% western blotting reagent and 0.02% sodium azide). We used monoclonal mouse anti-GST, sc-138, (Santa Cruz), diluted 1∶500 and polyclonal goat anti-human Ma2, sc-68099 (Santa Cruz), diluted 1∶1000 as positive controls. The polyclonal goat anti-human antibody specifically recognizes Ma2 and cannot detect Ma1. We used serum samples from two healthy donors with Ma2 autoantibody titer below cutoff (1900 AU), from two patients with primary SI-NET expressing Ma2 autoantibodies below cutoff and two patients with primary SI-NET expressing Ma2 autoantibodies at higher titer than cutoff. All the serum samples were diluted 1∶50. After the incubation, each strip was washed with TBS, 0.1% Tween-20 and incubated with secondary antibodies HRP-rabbit anti-mouse, P0161, diluted 1∶5000, HRP-donkey anti-goat, sc-2020, diluted 1∶5000, and HRP-rabbit anti-human IgG, P0214, diluted 1∶3000. The strips were then washed, incubated 10 seconds with Lumi-Light Western Blotting Substrate (Roche) and exposed in a Biorad Chemidoc XRS System (Bio-Rad Laboratories, Hercules, CA). The image was elaborated by using Quantity One software.

### *In vitro* transcription-coupled translation and sequential immunoprecipitation

^35^S Met–radiolabeled human PNMA2 protein was produced by *in vitro* transcription coupled translation from the full-length cDNA clone for human PNMA2 (BioScience Geneservice) by using ^35^S-methionine and TnT® SP6 Quick Coupled Transcription/Translation System (Promega), according to manufacturer's instructions. During sequential immunoprecipitation two independent immunoprecipitations were performed. Briefly, in the first immunoprecipitation, serum samples were incubated with 90,000 counts per minute (CPM) ^35^S-labeled PNMA2. Positive and negative controls were incubated with or without 0.5 µg of commercial polyclonal goat anti-human PNMA2. The final volume of each reaction was 50 µl by using the IP buffer (50 mmol/l NaCl, 20 mmol/l pH 7.4 Tris-HCl, 0.02% sodium azide, 0.1% BSA and 0.15% Tween-20) used both for washing and dilution steps. Incubation was performed at 4°C O.N. The day after 50 µl of protein G-Sepharose beads (GE Lifescience) were incubated to each sample. After incubation at 4°C for 2 hours the samples were centrifuged at 380 *g*, the beads were collected and the supernatants were removed. The beads were washed 5 times, by using IP buffer and centrifugations. Then, 50 µl of IP buffer was added to each sample. The beads were then heated at 80°C for 5 minutes and the proteins were released. After centrifugation the supernatants were collected. For the second immunoprecipitation 0.6 µg of goat anti-human Ma2 antibody, previously used in the western blot analysis, was added to the new samples and incubated at 4°C, O.N. Thereafter, we added 50 µl protein G-Sepharose beads to each sample and continued the incubation at 4°C for 2 hours. The samples were centrifuged at 380 *g*, and washed 5 times with IP buffer to collect the beads. They were resuspended in 40 µl of SDS-PAGE 4X loading buffer containing 5% β-mercaptoethanol. The immunocomplexes, containing ^35^S-labeled PNMA2 protein, were released and denatured from the beads by heating at 95°C for 5 minutes. After centrifugation 20 µl of each sample were resolved by 10% SDS-PAGE. The gel was dried, subjected to autoradiography, by using phosphoimager 425S (Molecular Dynamics, Sunnyvale, CA) and analyzed by using ImageQuant software.

### Immuno-(Dot)-Blot for the detection of the anti-Ma2 and anti-Ma1

Recombinant immunoblot was used to detect the anti-Ma2 and anti-Ma1 antibodies. We used the available strips, spotted with the recombinant proteins. RAVO reagents were used for staining the recombinant proteins in the positive control strip according to the manufacturer's instruction. We also used 5 different strips for evaluating commercial antibodies and patient sera by using the method described above in Material & Methods (Western Blot Analysis) to use the same reagents and protocols to evaluate both commercial antibodies and sera. We used rabbit anti-PNMA2 (Atlas Antibodies) and goat anti-human Ma2 (SantaCruz) 1∶1000 to verify the specificity of these commercial antibodies. Serum samples from one healthy donor with low titer of anti-Ma2 and one serum sample from one patient with high titer of anti-Ma2. Serum samples were diluted 1∶10. Primary Antibodies and serum samples were incubated at 4°C over night. We used HRP-goat anti-rabbit (1∶3000, Dako), HRP-donkey anti-goat (1∶3000, SantaCruz) and HRP-rabbit anti-human IgG (1∶3000, Dako) as secondary antibodies. The strips were then washed, incubated 10 s with Lumi-Light Western Blotting Substrate (Roche) and exposed in a Biorad Chemidoc XRS System (Bio-Rad Laboratories, Hercules, CA), as described in Western Blot Analysis.

## Supporting Information

Figure S1 **Immuno-(dot)-blot used to detect anti-Ma2 and anti-Ma1.** Positive control (lane 1), Negative control (lane 2), Goat anti-Ma2 antibody specifically detects Ma2 (lane 3), Rabbit anti-Ma2 antibody specifically detects Ma2 (lane 4), Serum from healthy donor fails to detect Ma2 (lane 5) whereas Serum from one SI-NET patient with high titer of Ma2 autoantibodies detects Ma2 and faintly Ma1 (lane 6). Reaction control evaluates the performance of the blot.
(TIF)

Figure S2 **Immunostaining of Ma2 on specimens from untreated SI-NET patients matched with blood samples.** 12 patients out of 12 expressing high titer of Ma2 autoantibodies, as described in Table S1, were positively stained. Eight patients with low titer of Ma2 autoantibodies, as described in Table S1, showed that 7 out of 8 specimens were positively stained while one was negative. Figure S2 shows four representative staining from patients with high titer of Ma2 autoantibodies, panel A and with low titer of Ma2 autoantibodies, panel B. Bar  = 50 µm.
(TIF)

Figure S3 **Serum from primary SI-NET patients with high anti-Ma2 titer efficiently immunostain tumor cells and neurons on tissue sections from primary SI-NETs.** We stained paraffin embedded tissue sections from untreated primary SI-NET patients, by using serum from a primary SI-NET patient with high anti-Ma2 titer and serum from a healthy donor. One representative Ma2 staining is shown. In upper panels, on the left tumor cells and on the right neurons, located in the Auerbach's plexus (or myenteric plexus) are Ma2 stained. In lower panels, serum from healthy donor faintly stains Ma2. Tumor cells are shown on the left and neurons on the right. Bar  = 50 µm.
(TIF)

Figure S4 **Ma2 Immunoreactivity in lung carcinoids.** The mean of Ma2-positive tumor cells in typical carcinoids is 54% and in atypical carcinoids is 28%, independent of tumor growth patterns of the former. Typical carcinoids exhibited either diffuse positivity of tumor cells in trabecular growing tumors A and as an inset A-bis or heterogeneous distribution of the signal in spindle cell tumors or spindle cell component of tumors B. Atypical carcinoids presented with heterogeneous distribution of the immunostaining product inside tumor cells with intermingling of negative and faint to moderate reactivity C or completely negative tumor cells D. Bar  = 50 µm.
(TIF)

Table S1 Circulating Ma2 autoantibody levels in serum samples and cytoplasmic Ma2 expressions in paraffin-embedded tissues of 20 SI-NET patients.
(DOC)
