# Supplementary figures and images for: Paraneoplastic Antigen Ma2 Autoantibodies as Specific Blood Biomarkers for Detection of Early Recurrence of Small Intestine Neuroendocrine Tumors
Source: PLoS One. 2010 Dec 30;5(12):e16010. doi: 10.1371/journal.pone.0016010 (PMC3012732; doi:10.1371/journal.pone.0016010)

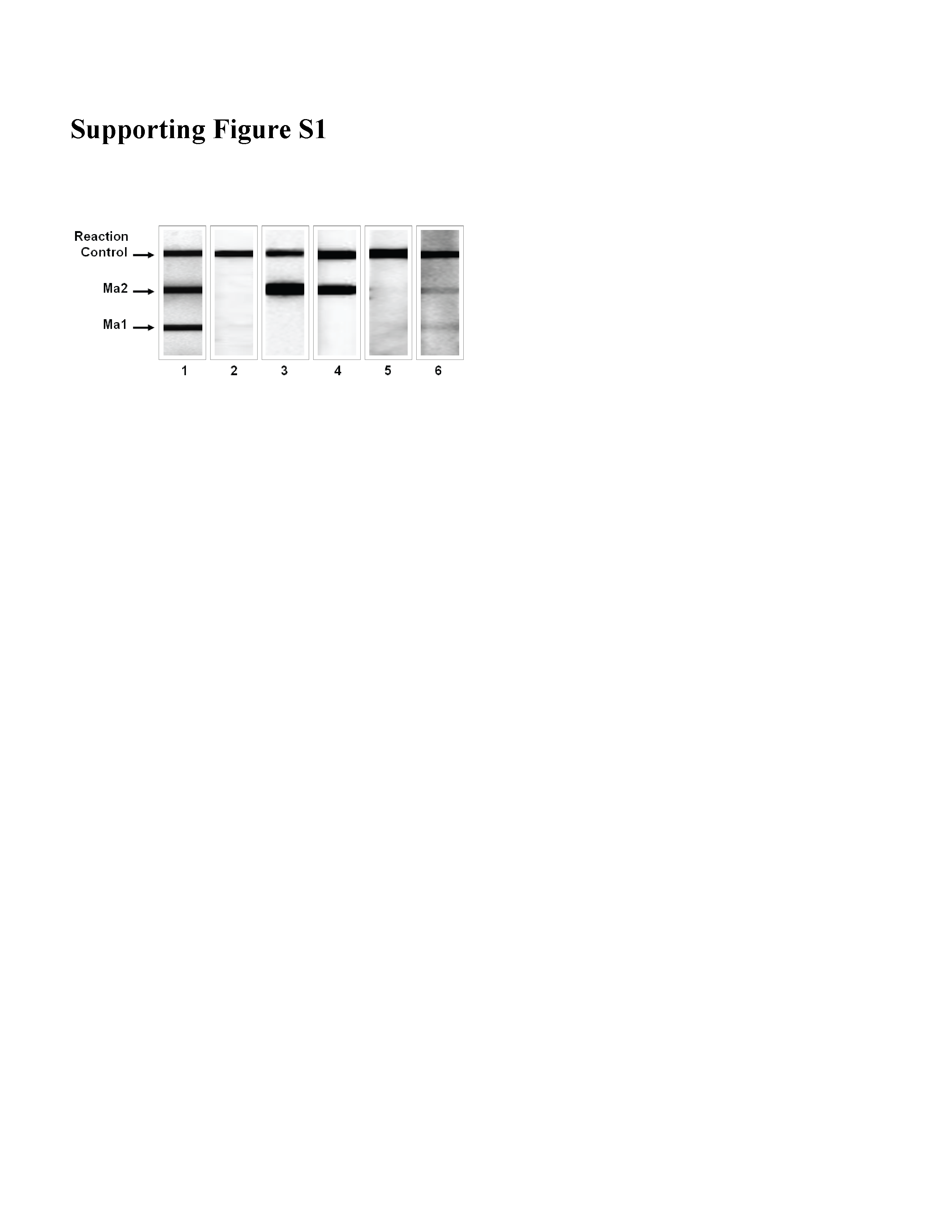

Supplement: Figure S1 — Immuno-(dot)-blot used to detect anti-Ma2 and anti-Ma1. Positive control (lane 1), Negative control (lane 2), Goat anti-Ma2 antibody specifically detects Ma2 (lane 3), Rabbit anti-Ma2 antibody specifically detects Ma2 (lane 4), Serum from healthy donor fails to detect Ma2 (lane 5) whereas Serum from one SI-NET patient with high titer of Ma2 autoantibodies detects Ma2 and faintly Ma1 (lane 6). Reaction control evaluates the performance of the blot. (TIF) [file pone.0016010.s001.tif]

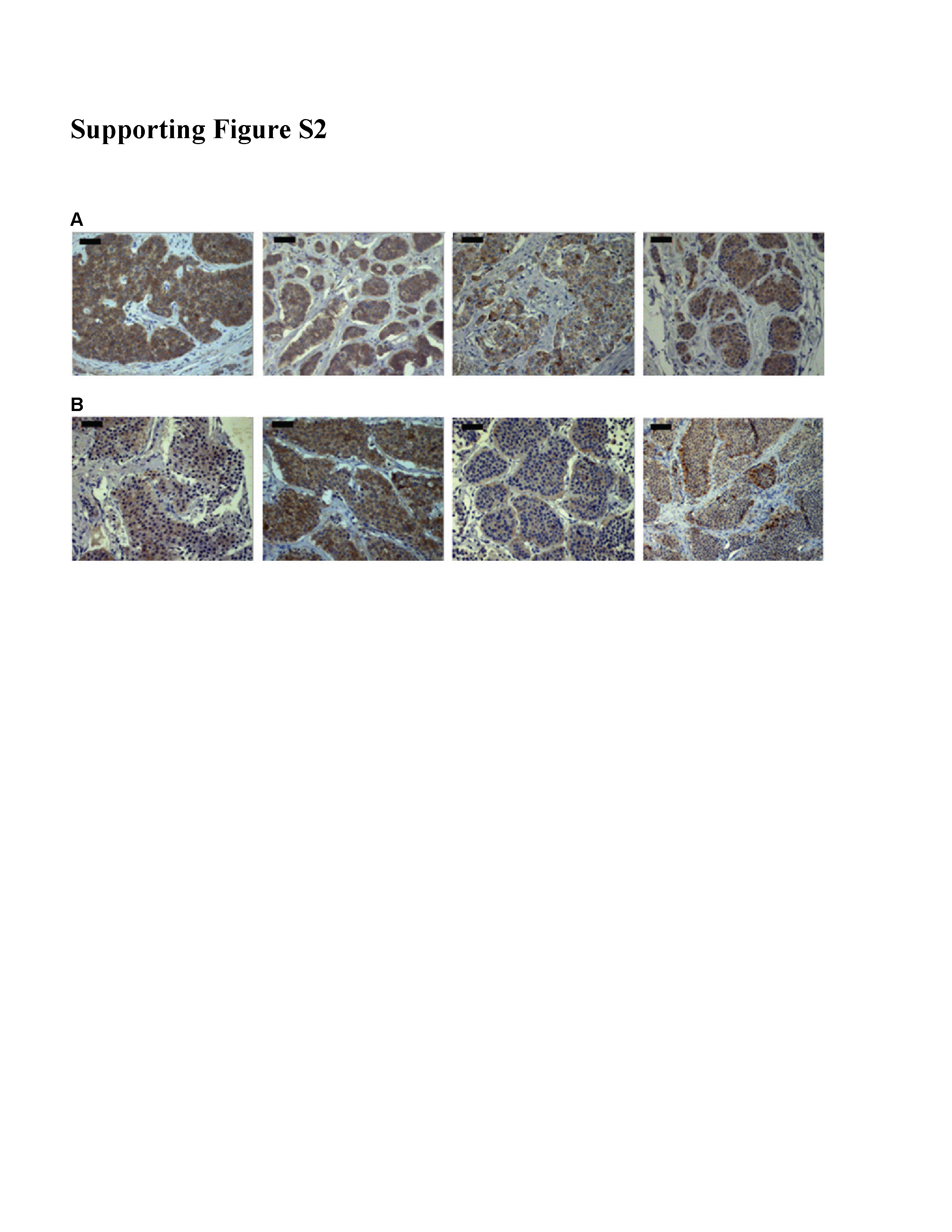

Supplement: Figure S2 — Immunostaining of Ma2 on specimens from untreated SI-NET patients matched with blood samples. 12 patients out of 12 expressing high titer of Ma2 autoantibodies, as described in Table S1, were positively stained. Eight patients with low titer of Ma2 autoantibodies, as described in Table S1, showed that 7 out of 8 specimens were positively stained while one was negative. Figure S2 shows four representative staining from patients with high titer of Ma2 autoantibodies, panel A and with low titer of Ma2 autoantibodies, panel B. Bar = 50 µm. (TIF) [file pone.0016010.s002.tif]

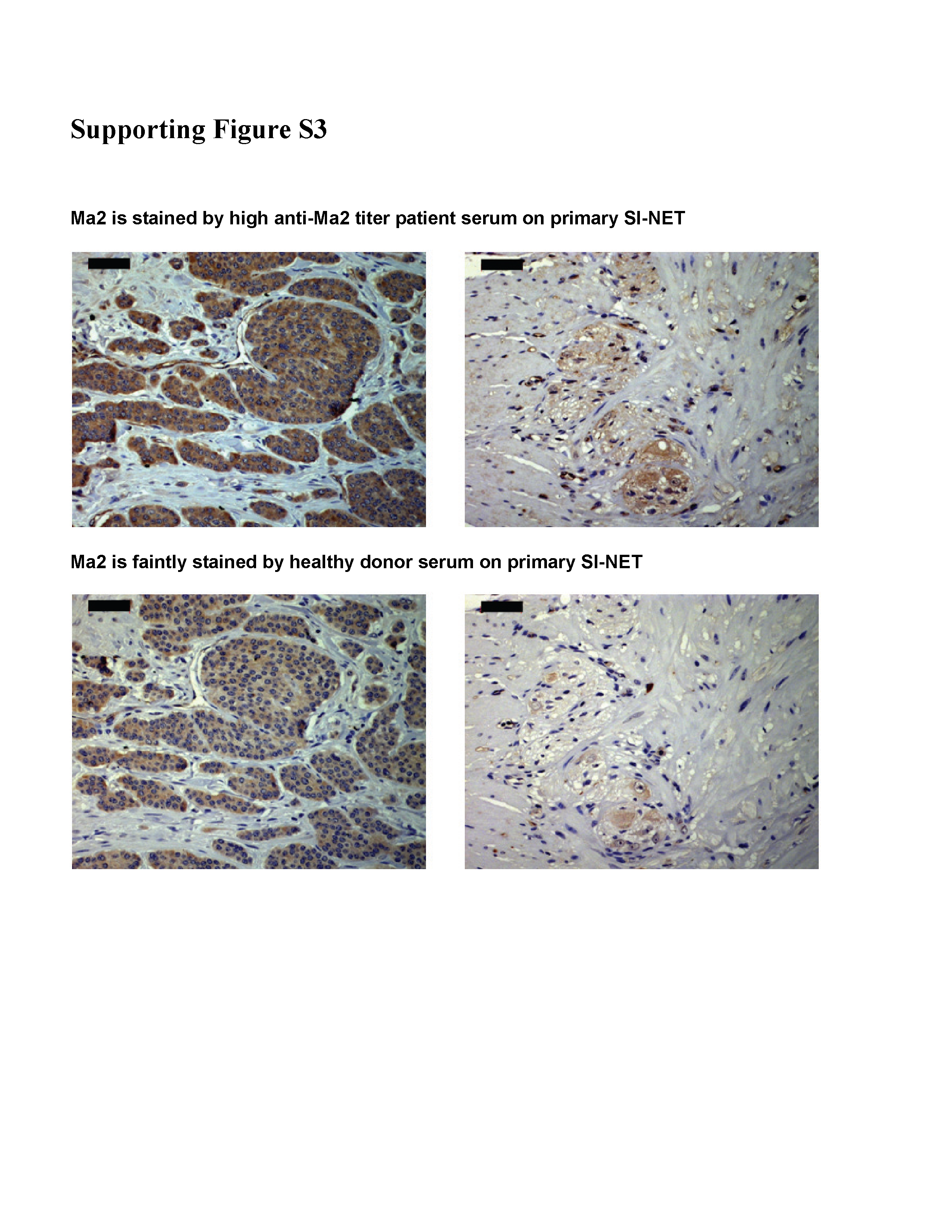

Supplement: Figure S3 — Serum from primary SI-NET patients with high anti-Ma2 titer efficiently immunostain tumor cells and neurons on tissue sections from primary SI-NETs. We stained paraffin embedded tissue sections from untreated primary SI-NET patients, by using serum from a primary SI-NET patient with high anti-Ma2 titer and serum from a healthy donor. One representative Ma2 staining is shown. In upper panels, on the left tumor cells and on the right neurons, located in the Auerbach's plexus (or myenteric plexus) are Ma2 stained. In lower panels, serum from healthy donor faintly stains Ma2. Tumor cells are shown on the left and neurons on the right. Bar = 50 µm. (TIF) [file pone.0016010.s003.tif]

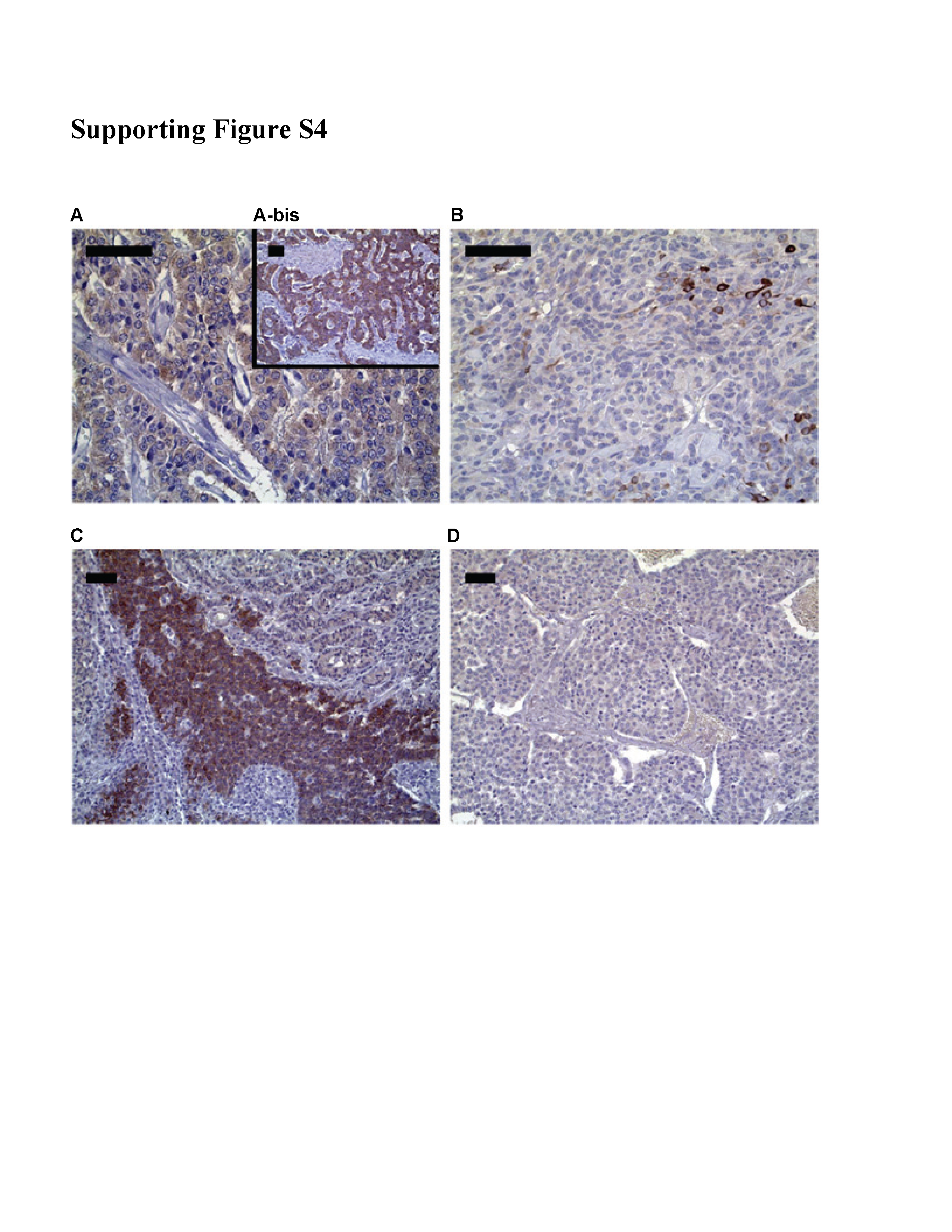

Supplement: Figure S4 — Ma2 Immunoreactivity in lung carcinoids. The mean of Ma2-positive tumor cells in typical carcinoids is 54% and in atypical carcinoids is 28%, independent of tumor growth patterns of the former. Typical carcinoids exhibited either diffuse positivity of tumor cells in trabecular growing tumors A and as an inset A-bis or heterogeneous distribution of the signal in spindle cell tumors or spindle cell component of tumors B. Atypical carcinoids presented with heterogeneous distribution of the immunostaining product inside tumor cells with intermingling of negative and faint to moderate reactivity C or completely negative tumor cells D. Bar = 50 µm. (TIF) [file pone.0016010.s004.tif]
