# Supplementary material for: Paraneoplastic Antigen Ma2 Autoantibodies as Specific Blood Biomarkers for Detection of Early Recurrence of Small Intestine Neuroendocrine Tumors
Source: PLoS One. 2010 Dec 30;5(12):e16010. doi: 10.1371/journal.pone.0016010 (PMC3012732; doi:10.1371/journal.pone.0016010)
Supplement: Table S1 — Circulating Ma2 autoantibody levels in serum samples and cytoplasmic Ma2 expressions in paraffin-embedded tissues of 20 SI-NET patients. (DOC) [file pone.0016010.s005.doc]

**Supporting Table S1**

**Supporting Table S1**  Circulating Ma2 autoantibody levels in serum samples and cytoplasmic Ma2 expressions in paraffin-embedded tissues of 20 SI-NET patients

| **Patient** | **Sex/age*^a^*** | **Treatment** | **Tumor Type*^b^*** | **Serum Anti-Ma2 (AU) *^c^*** | **Tissue Ma2 Immunostaining** |
| --- | --- | --- | --- | --- | --- |
| 1 | M/67 | Untreated | LNM | 2856 ( > cutoff) | + |
| 2 | M/57 | Untreated | LNM | 2531 ( > cutoff) | + |
| 3 | M/59 | Untreated | LM | 2218 ( > cutoff) | + |
| 4 | M/69 | Untreated | LM | 2108 ( > cutoff) | + |
| 5 | F/67 | Untreated | LM | 2110 ( > cutoff) | + |
| 6 | F/54 | Untreated | P | 3794 ( > cutoff) | + |
| 7 | M/59 | Untreated | LNM | 2641 ( > cutoff) | + |
| 8 | F/62 | Untreated | LM | 3282 ( > cutoff) | + |
| 9 | M/65 | Untreated | P | 2098 ( > cutoff) | + |
| 10 | F/68 | Untreated | LM | 1856 ( > cutoff) | + |
| 11 | M/34 | Untreated | LM | 2194 ( > cutoff) | + |
| 12 | F/61 | Untreated | LM | 2778 ( > cutoff) | + |
| 13 | M/60 | Untreated | LNM | 1712 ( < cutoff) | + |
| 14 | M/74 | Untreated | LM | 882 ( < cutoff) | + |
| 15 | F/67 | Untreated | LM | 671 ( < cutoff) | + |
| 16 | M/52 | Untreated | LM | 1484 ( < cutoff) | + |
| 17 | F/72 | Untreated | LM | 972 ( < cutoff) | + |
| 18 | F/75 | Untreated | LNM | 1852 ( < cutoff) | + |
| 19 | F/69 | Untreated | LM | 1297 ( < cutoff) | + |
| 20 | F/71 | Untreated | LM | 591 ( < cutoff) | – |

***^a^*** M, male; F, female; Age at the time of operation

***^b^*** P, primary tumor; LNM, lymph node metastasis; LM, liver metastasis

***^c^*** AU, arbitrary unit; cutoff, 1900AU chosen for the indirect ELISA data
